# Psychological Maltreatment, Trait Mindfulness, and Marital Quality: An Actor–Partner Interdependence Model

**DOI:** 10.1007/s11126-025-10171-5

**Published:** 2025-06-07

**Authors:** Süleyman Akçıl

**Affiliations:** Zonguldak Bülent Ecevit University, Ereğli Faculty of Education, Ereğli, Zonguldak, Turkey

**Keywords:** Psychological maltreatment, Mindfulness, Relationship quality, Actor-partner interdependence model, Dyadic study

## Abstract

Each partner’s unique experiences may shape the quality of their romantic relationship. Individuals with a history of psychological maltreatment may need to exercise greater mindfulness to preserve relational well-being. This study examined the mediating role of mindfulness in marriage in the association between psychological maltreatment and relationship quality, considering both actor and partner effects. The Actor–Partner Interdependence Mediation Model (APIMeM) was used to analyze data from 361 married couples (*N* = 722). The average age was 35.39 years (SD = 8.29) for men and 33.02 years (SD = 7.83) for women. Participants completed standardized self-report measures of psychological maltreatment, marital mindfulness, and relationship quality. Results showed that mindfulness significantly mediated the relationship between psychological maltreatment and relationship quality at both the actor and partner levels. In partner effects, one partner’s experience of maltreatment predicted the other’s relationship quality through the latter’s mindfulness. These findings suggest that higher levels of mindfulness—both individual and partner-based—may buffer the negative effects of psychological maltreatment on relationship quality.

**Supplementary Information:**

The online version contains supplementary material available at 10.1007/s11126-025-10171-5.

## Introduction

Every individual forms social connections that qualify as close relationships. Finkel et al. define a close relationship as one involving emotional attachment and interdependence, enabling individuals to meet essential needs and achieve goals [12]. Intimate relationships help satisfy social and psychological needs. Relationship quality refers to how positively or negatively individuals evaluate their relationships, regardless of type (e.g., friendships, dating, or marriage). It plays a significant role because subjective evaluations shape partner perceptions [36]. Individual perceptions and expectations influence relationship satisfaction. Therefore, relationship quality is key to positive relational experiences. Adults often regard romantic partners and close friends as central figures in their social networks [4]. Romantic relationships—defined as long-term partnerships including dating, cohabitation, or marriage [24]—are consistently linked to greater life satisfaction and mindfulness [34, 39]. Those reporting higher romantic relationship quality also show lower levels of suicidal ideation [41]. These associations highlight the mental health benefits of high-quality romantic bonds. Farooqi suggests that relationship quality depends on joint interaction, emotional sharing, and beliefs about romance [10]. Romantic relationships are thus considered critical to psychological well-being and quality of life [9, 27].

Men develop new connections in their environments, such as their spouses’ acquaintances and family, through marriage, which is a form of romantic relationship. These connections can offer social support. However, social support does not always lead to social integration [41]. Therefore, relationship quality is especially significant in marriage. According to Colman and Widom, individuals who experienced childhood maltreatment had significantly higher rates of cohabitation, walking out, and divorce than those who did not [5]. Such experiences impair their ability to establish and maintain healthy romantic relationships in adulthood. Childhood maltreatment—**including physical and emotional abuse, sexual abuse, neglect, and exploitation in a relationship of responsibility or trust—**can harm a child’s development, health, and dignity [44]. It is believed that such maltreatment initiates maladaptive developmental processes, increasing the likelihood of mental health and romantic relationship difficulties later in life [13]. For both genders, early maltreatment is associated with lower relationship quality [45]. These long-term consequences highlight how early trauma affects adult relational functioning. Thus, understanding its impact may require going beyond individual-level research.

Given the potential for childhood maltreatment to influence marital and romantic relationships, it is important to prevent or mitigate its adverse effects. Mindfulness may help individuals process past trauma and foster healthy, satisfying relationships. Defined as intentional attention to present-moment internal and external experiences, mindfulness is commonly cultivated through meditation [3]. Both online and in-person mindfulness practices have shown positive effects on mental health indicators such as anxiety, depression, and stress [23, 38]. Moreover, mindfulness contributes to relationship satisfaction and overall well-being within couples [43]. For example, mindfulness, reduced tension, and positive relational behaviors are predictors of relationship quality [35]. Thus, both individual mindfulness and dyadic processes are critical for maintaining healthy relationships. Although stress can disrupt intimacy, mindfulness and constructive behaviors buffer these effects and enhance relationship quality. Research using longitudinal and dyadic methods has examined how mindfulness relates to perceived partner responsiveness and relational dynamics [40]. These approaches help assess the long-term role of mindfulness in shaping partner perceptions and interactions. One dyadic study found that both partners’ mindfulness and early adverse experiences predicted their perceived relationship quality. This suggests that individuals’ past experiences influence how they approach current romantic relationships. Mindfulness can promote emotional attunement, conflict resolution, and deeper relational understanding within marriage.

## The Present Study

A fundamental human requirement is the establishment of positive intimate relationships [7]. It is only natural that the mental health of both partners in a romantic relationship is influenced by—and in turn affects—the functioning of the relationship, given the central role of close relationships in most individuals’ lives [11]. Furthermore, the quality of romantic relationships, defined as the degree to which individuals evaluate them positively or negatively, significantly shapes the relationship’s future trajectory [10]. The literature emphasizes the importance of examining positive aspects of romantic relationships, including social support, healthy communication, and relationship satisfaction [11]. Accordingly, romantic relationship quality is the main focus of the present study, as its impact on well-being exceeds that of simply being in a relationship [26]. Given this, it is understandable that individuals desire high-quality marriages. Moreover, individuals’ past experiences may affect their later romantic functioning. In particular, earlier negative experiences can threaten marital relationships. According to the Couple Adaptation to Traumatic Stress Model (CATS), trauma can create a cycle of mutual stress, affecting both partners [22]. In this context, mindfulness may help mitigate trauma’s negative effects by supporting conscious communication and healthier stress management. Consequently, the present study aims to examine the mediating role of mindfulness in the relationship between childhood maltreatment and romantic relationship quality in married couples. The Actor-Partner Interdependence Model (APIM) allows for the examination of how an individual’s experiences affect both their own outcomes (actor effects) and their partner’s outcomes (partner effects) [29]. Thus, the study investigates the proposed relationships and hypotheses within a dyadic framework.

*RQ1*. In the context of actor effect, does mindfulness in marriage play a mediating role in the relationship between psychological maltreatment and relationship quality in female and male?

*RQ2*. Does mindfulness in marriage have a mediating role in the relationship between psychological maltreatment and relationship quality in couples in the context of partner influence?

### Methods

#### Participants and Procedure

A total of 722 individuals, including 361 Turkish heterosexual married couples, participated in the study. The mean age of the female participants was 33.02 years (SD = 7.83), and the mean age of the male participants was 35.39 years (SD = 8.29). 30.2% of these couples had been married for at least 10 years. 60.7% of them had at least 1 child. 65.1% of both men and women were at least university graduates. Most of the participants, 85.6% of women and 83.9% of men, indicated their perceived socio-economic level as “medium”.

The sample size was guided by previous dyadic studies using the Actor–Partner Interdependence Model. Studies have suggested that approximately 300 to 350 dyads are sufficient to detect small-to-medium actor and partner effects with acceptable power in mediation models [46]. In line with this, the current sample of 361 couples (*N* = 722) was considered sufficient for the planned analyses.

The study collected data from volunteer participants via social media. Individuals had to be married and provide informed consent to participate in the study. This investigation adhered to the principles of the 1975 Declaration of Helsinki, which underwent revision in 2000. The [Omitted] University Scientific Research and Ethical Review Board granted approval for the investigations (Report Number: XXX).

### Measures

#### Perceived Relationship Quality Scale

It was developed by Fletcher et al. to assess the perceived quality of the overall romantic relationship [19]. The adaptation study of the scale into Turkish was conducted by Sağkal and Özdemir [39]. Cronbach’s alpha coefficient was found to be 0.86 in the analyzes conducted to determine the reliability of the scale in the adaptation study. The scale includes one dimension and 6 items. It is a 7-point Likert-type scale and is graded between 1 (not at all) and 7 (very much). The highest score that can be obtained from the scale is 6, and the lowest score is 42. A higher score on the scale indicates that the quality of the romantic relationship is generally perceived as better.

#### Mindfulness in Marriage Scale

This scale was developed by Erus and Deniz to determine the level of interpersonal mindfulness in marital relationships [8]. The calculated Cronbach’s alpha internal consistency coefficients were 0.87 and 0.85. The scale has 12 items and has a unidimensional structure. The answer options are in 5-point Likert type and are graded between 1 (Never) and 5 (Always). The highest score that can be obtained from the scale is 60, and the lowest score is 12. A high score on the scale is considered to indicate that the individual has a high level of interpersonal mindfulness in his/her relationship and communication with his/her spouse.

#### Psychological Maltreatment Questionnaire

It was developed in Arslan to measure childhood psychological abuse experiences [2]. The Cronbach alpha internal consistency coefficient calculated in the development study of the scale is 0.95. The 12-item short form of the scale was used in this study. It includes 2 dimensions consisting of 12 items in total. The response options are 4-point Likert type and are graded between 1 (Never) and 4 (Always). The highest score is 48, and the lowest score is 12. As the scores increase, it is understood that the level of psychological abuse is high.

### Data Analysis

This study examined how psychological maltreatment through mindfulness can predict the quality of relationships in married couples. A reciprocal-relational model was employed to evaluate the direct and indirect effects of couples on each other, as well as their effects on themselves.

Initially, the data obtained from the pairings were matched. Consequently, the final data set was obtained. IBM SPSS Statistics 23 to conduct preliminary analyses, including reliability, descriptive, and correlational analyses were employed (Table 1). The structural model was subsequently tested using path analysis, and binary analysis techniques were implemented using AMOS. The analysis included the implementation of the item-parceling technique in single-factor assessments [33]. Consequently, item-parceling was implemented since mindfulness and relationship quality were unidimensional in SEM. This method is appropriate for the application of personality trait concepts, as it reduces the number of observed variables, enhances reliability, and facilitates the display of a normal distribution on the scales [37]. To assess the overall fit of the data, a variety of fit indices, including goodness of fit index (GFI), comparative fit index (CFI), tucker-lewis index (TLI), incremental fit index (IFI), normed fit index (NFI), and standardized root mean square residual (SRMR), were applied [25]. APIM, a binary analysis approach, and the Actor-Partner Interaction Mediation Model (APIMeM), an extension of APIM, were implemented.

## Results

### **Descriptive Statistics**,** Gender Differences**,** and Correlations**

Table 1 illustrates the descriptive statistics of the variables examined in the investigation and the interrelationships among them. The examination of the actor relationships between the variables reveals that the quality of their relationships and mindfulness in marriage are positively correlated for both men and women (Female *r* =.65, *p* <.01; Male *r* =.67, *p* <.01). Conversely, psychological maltreatment was negatively associated with both genders’ mindfulness in marriage (Female: *r* = −.20, *p* <.01; Male: *r* = −.29, *p* <.01) and relationship quality (Female: *r* = −.26, *p* <.01; Male: *r* = −.27, *p* <.01).

**Table 1:** Descriptive statistics and reliabilities for the study variables

Variable	1	2	3	4	5	6
1. Maltreatment (Female)	–					
2. Maltreatment (Male)	0.30**	–				
3. Relationship Quality (Female)	− 0.26**	− 0.19**	–			
4. Relationship Quality (Male)	− 0.14**	− 0.27**	0.46**	–		
5. Mindfulness (Female)	− 0.20**	− 0.21**	0.65**	0.45**	–	
6. Mindfulness (Male)	− 0.26**	− 0.29**	0.42**	0.67**	0.45**	–
Mean	19.72	19.69	35.82	37.07	50.75	49.85
SD	6.31	6.24	5.95	5.94	6.43	6.95
Skewness	1.17	1.04	-1.09	-1.72	− 0.868	− 0.767
Kurtosis	1.98	1.12	0.801	3.13	0.804	0.361
McDonald ω	0.889	0.878	0.910	0.919	0.825	0.837
Cronbach α	0.884	0.872	0.896	0.911	0.825	0.837
Guttmann λ6	0.907	0.917	0.894	0.924	0.866	0.880

### Actor-Partner Independence Mediator Model (APIMeM)

Initially, the measurement models based on data from both men and women were evaluated separately and jointly. The model included three latent variables—psychological maltreatment, mindfulness in marriage, and relationship quality—and six observed variables. The measurement models demonstrated a satisfactory fit when the data from women and men were analyzed separately (χ² (6, *N* = 361) = 1.335; RFI = 0.98; GFI = 0.99; NFI = 0.99; IFI = 0.99; TLI = 0.99; RMSEA = 0.03; SRMR = 0.01; χ2 (6, *N* = 361) = 1.268; RFI = 0.98; GFI = 0.99; NFI = 0.99; IFI = 0.99; TLI = 0.99; RMSEA = 0.02; SRMR = 0.01. Finally, the measurement model that was used to evaluate the data from both men and women demonstrated a satisfactory fit; χ2 (39, *N* = 361) = 1.737; RFI = 0.95; GFI = 0.97; NFI = 0.97; IFI = 0.98; TLI = 0.97; RMSEA = 0.04; SRMR = 0.02.

**Fig. 1:**
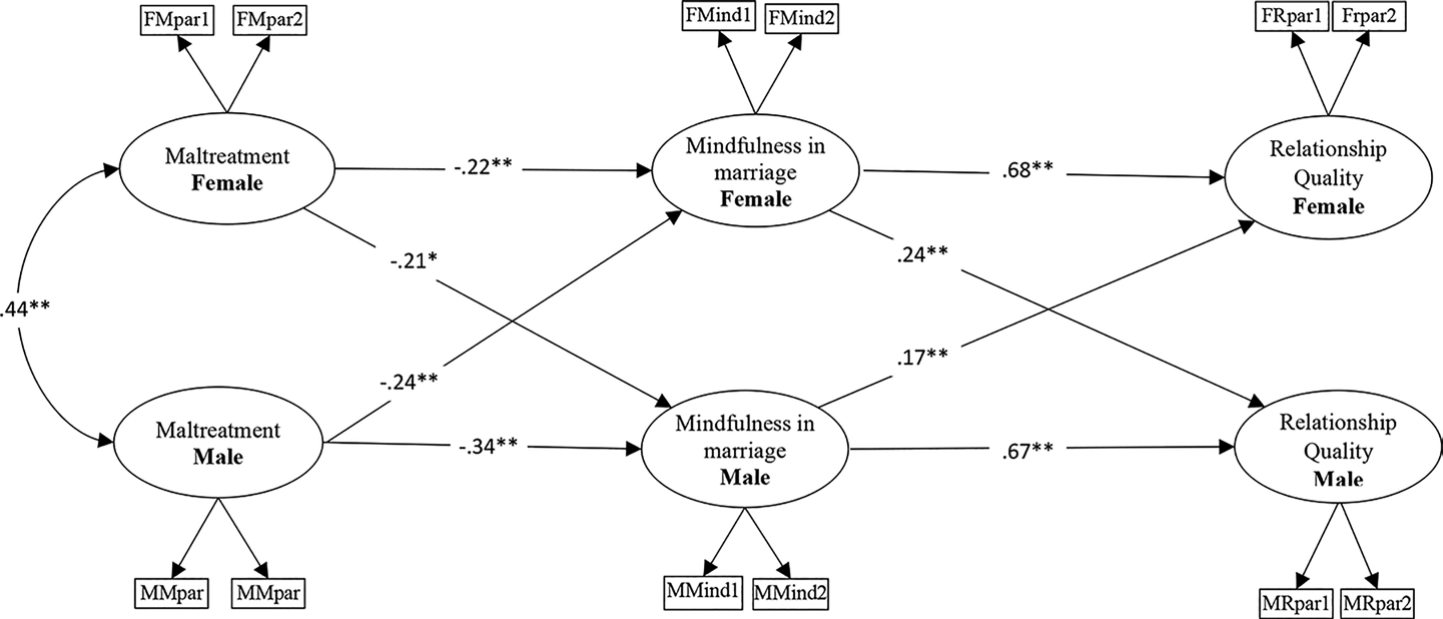
Actor-partner independence mediator model. Note. *N* = 722; ^*^p <.05, ^**^p <.01; FMpar parcels of female’s psychological maltreatment; MMpar parcels of male’s psychological maltreatment; FMind parcels of female’s mindfulness in marriage; MMind parcels of male’s mindfulness in marriage; FRpar parcels of female’s relationship quality; MRpar parcels of male’s relationship quality

The mediator function of mindfulness in marriage in the relationship between psychological maltreatment and relationship quality in couples was tested using the actor-partner interdependence mediation model, in accordance with the measurement model. χ2 (45, *N* = 361) = 3.318; RFI = 0.90; GFI = 0.93; NFI = 0.93; IFI = 0.95; TLI = 0.93; RMSEA = 0.08; SRMR = 0.08; and the fit indices of the model were satisfactory. All actor and partner paths yielded statistically significant effects. Figure 1 illustrates the structural model and standardized path coefficients. The model examines how a partner’s mindfulness in marriage mediates the link between psychological maltreatment and relationship quality, independent of gender. It incorporates both actor-partner effects and mediating actor-partner effects.

## Discussion

Researchers may conduct individual studies to strengthen the family structure, but they can also support couples in resolving their conflicts. Particularly, the psychological maltreatment individuals endure during childhood may shape the quality of romantic relationships they establish in adulthood. The emotional gratification that an individual experiences from a relationship is referred to as “romantic relationship quality.” This multidimensional concept includes various components, such as commitment, loyalty, trust, intimacy, passion, and love [19]. Low-quality romantic relationships can negatively impact an individual’s mental health [41].

The Couple Adaptation to Traumatic Stress (CATS) model provides a framework for understanding how maltreatment affects relationship quality. It also emphasizes that personal, interpersonal, and community resources can buffer the detrimental effects of maltreatment on relationships [22]. Based on this theoretical framework, the current study examined the mediating role of mindfulness in the relationship between maltreatment and relationship quality. The relationship between these concepts in married couples was investigated using APIM as a dyadic method. The Actor-Partner Interdependence Model (APIM) allows for analysis of how maltreatment, mindfulness, and relationship quality relate for both partners—capturing actor effects (how one’s experiences affect their own outcomes) and partner effects (how they influence their partner’s outcomes) [28].

The relationships between these concepts have been investigated at the individual level in previous research, but not often within a dyadic context. To gain deeper insight, dyadic studies that account for couple dynamics should be carried out. Accordingly, this investigation is among the first to explore the mediating role of mindfulness in the relationship between maltreatment and the quality of romantic relationships from a dyadic perspective. Hypotheses were developed based on existing literature to guide the investigation.

The analyses conducted for the initial hypothesis indicated that mindfulness acts as a mediator between maltreatment and relationship quality within the context of actor effects. This finding has implications for both men and women. Individuals with histories of peer abuse, sexual abuse, or sibling maltreatment tend to report lower mindfulness levels [30]. Depressive symptoms and maladaptive rumination may partially explain the link between childhood maltreatment and mindfulness [20]. Lower mindfulness has also been associated with increased depression, anxiety, dissociation, sleep disturbances, sexual difficulties, and PTSD symptoms [15].

Those exposed to childhood maltreatment often report reduced relationship mindfulness, which is linked to more negative and less positive relationship experiences [16]. Along similar lines, Fitzgerald et al. showed that the sequential indirect path from maltreatment to relationship quality—via mindfulness and post-traumatic stress symptoms—was substantial [18]. In addition, mindfulness appears to moderate the association between maltreatment and various marital outcomes, such as support, distress, and overall relationship quality [17]. A longitudinal study also found that relationship quality mediates the link between childhood maltreatment and later symptoms of depression and social anxiety, suggesting that a satisfying romantic relationship may serve as a corrective emotional experience for adults with early adversity [14]. Taken together, these findings suggest that individuals with maltreatment histories are likely to show lower mindfulness, which, in turn, contributes to lower romantic relationship quality. Maintaining mindfulness, even in the aftermath of early negative experiences, appears essential for building and sustaining healthy romantic bonds.

The relationships between maltreatment, mindfulness, and romantic relationship quality were significant across genders in the current study. Gallo et al. report that childhood and adolescent physical and sexual abuse are risk factors for depression and anxiety in adulthood [21]. Although these effects may be more prominent among women, current evidence is not sufficient to confirm consistent gender differences. Alispahic and Hasanbegovic-Anic found gender-based variation in specific dimensions of mindfulness, highlighting the need to tailor mindfulness interventions accordingly [1]. Gender-sensitive approaches may help enhance relational outcomes more effectively. Larsen et al. showed that the frequency of childhood physical abuse had a significant impact on relationship quality for both men and women, but did not find any gender-based differences in this effect [31]. Taken together, while individual needs may differ during relational recovery, there appear to be common patterns in how maltreatment relates to romantic relationship quality across genders.

Analyses addressing partner effects were conducted to test the study’s second hypothesis. The results indicate that mindfulness mediates the relationship between maltreatment and relationship quality. This finding aligns with prior research. For instance, in a dyadic study of married couples, Cooper et al. found that both individuals’ and their partners’ relationship quality were predicted by mindfulness and adverse childhood experiences—regardless of gender [6]. While this supports the current findings, it’s worth emphasizing that the present study specifically focuses on mindfulness as a mediator within marriage in the link between maltreatment and relationship quality. Understanding mindfulness in this role may offer insight for interventions aimed at improving relationship quality among individuals with histories of maltreatment. High levels of mindfulness within marriage appear to support better relational outcomes, even when one or both partners have experienced early adversity. Recent systematic reviews also support the effectiveness of mindfulness-based couple interventions in promoting relational well-being and reducing distress [23, 43]. These reviews further reinforce the relevance of mindfulness in relational contexts, particularly when addressing trauma histories in couples. A study using APIM showed that each partner’s relationship quality was indirectly associated with their own maltreatment via self-compassion. However, self-compassion did not significantly predict their partner’s relationship quality [32]. Although partner effects were limited in that context, the current study found that maltreatment predicted relationship quality at both actor and partner levels when mindfulness was included. This suggests that, even in the presence of past trauma, quality relationships may be possible when one’s partner maintains high mindfulness.

### Limitations

The current study makes important contributions to the literature, but several limitations should be acknowledged. The first relates to data collection. Data were obtained through self-report from voluntary participants, which raises the possibility of social desirability bias or inaccurate reporting. In addition, the findings are limited by the specific measurement tools used. Future research could benefit from incorporating alternative methods such as peer reports, structured interviews, or behavioral observations to strengthen validity.

There is also a sampling limitation. As the sample consisted solely of married individuals living in Turkey, generalizing the findings should be done with caution. Studies involving individuals from different cultural backgrounds are needed to examine the universality of the results. Cultural norms related to marriage, emotional expression, and mindfulness may influence how the variables in the model interact and should be considered when interpreting the findings. Finally, the study’s methodological design presents a limitation. Although it used a dyadic approach, its cross-sectional nature prevents conclusions about causality. Longitudinal dyadic studies would allow for a more comprehensive understanding of the relationships between these variables over time. Although the current study examined the dyadic mediation of mindfulness in the relationship between psychological maltreatment and relationship quality, there may be other concepts such as emotional regulation, empathy, niceness that may has a role in romantic relationships. It is necessary to test different concepts as mediators in future research.

### Implications

This research aims to strengthen the institution of marriage by examining how mindfulness, maltreatment, and relationship quality are connected. The findings offer a clearer picture of marital dynamics and can inform the work of family and couple therapists, as well as mental health professionals. A meta-analysis conducted in 2012 found that couple therapy outperformed individual therapy in improving relationship satisfaction and yielded positive mental health outcomes [42]. A key contribution of this study is its focus on the role of mindfulness in the link between maltreatment and relationship quality. This suggests that mindfulness-based interventions may be especially beneficial for adults with histories of maltreatment [17]. Other research supports a broader approach, recommending that relationship interventions include not only mindfulness training but also stress reduction techniques and the promotion of positive couple behaviors [35]. Lastly, the findings advise policymakers to incorporate evidence-based interventions to foster mutual mindfulness and compassion as well as assessment instruments to look for early indicators of psychological abuse into preventive mental health efforts for couples. A better social culture that supports relational well-being can be developed by policies that encourage the spread of mindfulness training, such as by including it in public awareness campaigns or adult education programs.

To better understand the long-term influence of mindfulness on couple dynamics, future research should include longitudinal, dyadic, and multidimensional designs. Taken together, these findings may help guide mental health professionals in developing more effective relationship-focused interventions.

## Conclusion

The dyadic method was employed to examine actor and partner effects between maltreatment and romantic relationship quality. Mindfulness within marriage was tested as a mediator, and the relationships between the variables were found to be significant at both levels. The findings revealed that a partner’s level of maltreatment predicted not only their own mindfulness but also that of their spouse. In turn, mindfulness levels predicted the perceived quality of the relationship for both individuals. For spouses to accurately assess both their own and their partner’s relationship experiences, a high level of marital mindfulness appears essential. However, individuals with a history of maltreatment may evaluate their relationship—both self and partner aspects—more negatively, primarily due to diminished mindfulness in the relationship.

## Electronic Supplementary Material

Below is the link to the electronic supplementary material.

Supplementary Material 1

## Data Availability

Data will be available on request.

## Declarations

### Pre-registration Statement

This study was not pre-registered.
