# Supplementary material for: Psychological Maltreatment, Trait Mindfulness, and Marital Quality: An Actor–Partner Interdependence Model
Source: Psychiatr Q. 2025 Jun 7;97(1):63–74. doi: 10.1007/s11126-025-10171-5 (PMC13032991; doi:10.1007/s11126-025-10171-5)
Supplement: Supplementary file 1 — Supplementary Material 1 [file 11126_2025_10171_MOESM1_ESM.docx]

**Declaration of Competing Interests**

I declare that I have no known competing financial interests or personal relationships that could have appeared to influence the work reported in this paper.

Süleyman Akçıl, Ph.D.
